# Impact of Telemedicine on Asthma Control and Quality of Life in Children and Adolescents: A Systematic Review and Meta-Analysis

**DOI:** 10.3390/children12070849

**Published:** 2025-06-27

**Authors:** Julen Garcia Gerriko, Tregony Simoneau, Jonathan M. Gaffin, Marina Ortúzar Menéndez, Alejandro Fernandez-Montero, Laura Moreno-Galarraga

**Affiliations:** 1 Faculty of Science, UPNA, Universidad Pública de Navarra, 31008 Pamplona, Spain; garcia.134466@e.unavarra.es; 2 Division of Pulmonary Medicine, Boston Children’s Hospital, Harvard Medical School, Boston, MA 02115, USA; tregony.simoneau@childrens.harvard.edu (T.S.); jonathan.gaffin@childrens.harvard.edu (J.M.G.); 3 Pediatric Department, HUN, Hospital Universitario de Navarra, 31008 Pamplona, Spain; marina.ortuzar.menendez@navarra.es; 4 Occupational Medicine Department, Universidad de Navarra, 31008 Pamplona, Spain; afmontero@unav.es; 5 IdisNa, Instituto de Investigación Sanitaria de Navarra, 31008 Pamplona, Spain

**Keywords:** telemedicine, childhood asthma, adolescent asthma, quality of life, asthma control, asthma management

## Abstract

Background: Asthma is the most common chronic respiratory disease in children and adolescents, associated with high morbidity and healthcare costs. Telemedicine has emerged as a strategy to improve access to care, adherence to treatment and symptom control. However, its effectiveness in the pediatric population has not been clearly studied. Objective: To assess the clinical effectiveness of telemedicine interventions in asthma control and health-related quality of life in asthmatic children and adolescents. Methodology: A systematic review and meta-analysis were performed following PRISMA-2020 guidelines, with previous registration in PROSPERO (CRD42025251000837). Sixteen randomized clinical trials (n = 2642) with patients aged 2–18 years were included. The interventions included videoconferencing, mobile applications, web systems, interactive voice response and mobile units in schools. The outcomes were measured using validated quality-of-life (PAQLQ) and asthma control (ACT/c-ACT) questionnaires. Results: Seven studies were incorporated into the PAQLQ meta-analysis, whose overall effect was non-significant (mean difference = 0.06; 95% CI: −0.06 to 0.18; I^2^ = 5.7%). Five studies provided ACT/c-ACT data, showing a significant effect in favor of telemedicine (mean difference = 0.61; 95% CI: 0.32 to 0.90; I^2^ = 73%). Complementary qualitative analysis revealed improvements in adherence, reduction in exacerbations, emergency department visits and use of rescue medication. Conclusions: Telemedicine improves the clinical control of pediatric asthma, although its impact on the quality of life is limited. Multicenter trials with long-term follow-up and cost-effectiveness evaluation are required.

## 1. Introduction

Asthma represents a critical global health challenge, with important implications for morbidity, mortality, health resource use, economic impact and long-term health outcomes [1]. It is the most frequent chronic respiratory disease in the pediatric age group, with an increasing incidence and prevalence [2]. It is therefore a priority to develop effective and innovative therapeutic strategies, such as digital tools and telemedicine, which can contribute to better control of the disease.

Asthma is a chronic airway inflammatory disease characterized by recurrent episodes of breathing difficulty (dyspnea), coughing, wheezing and chest tightness. These symptoms are due to a variable airflow obstruction, usually reversible, and bronchial hyperresponsiveness to various triggers, such as allergens, infections, exercise, environmental pollutants or cold air. Although it can manifest at any age, it is especially frequent in childhood, and its severity and evolution are highly variable among patients [3,4]. Although its exact prevalence is difficult to estimate due to disparities in diagnostic methods and reporting systems, it is estimated that 1 in 10 children globally present asthma symptoms; approximately half of them experience inadequate disease control, and 10% meet the criteria for severe asthma [1]. In Spain, the International Study of Asthma and Allergies in Childhood (ISAAC) establishes wheezing prevalence levels of 9.9% in children aged 6–7 years and 10.6% in adolescents aged 13 and 14 years [5,6].

Asthma is becoming increasingly recognized as a complex and heterogeneous syndrome consisting of several different entities or phenotypes. The different asthma phenotypes present with varying severity of airway obstruction secondary to chronic inflammation [7,8]. The presence of symptomatology, limitation of physical activity and fear of exposure to various triggers are often associated with lower quality of life (QoL) [1]. Despite the importance of QoL in health and medicine, there is an ongoing conceptual and methodological debate about the meaning of QoL. There is no uniform definition of the concept; however, the World Health Organization (WHO) defines QoL as “an individual’s perception of his or her position in life in the context of the culture in which he or she lives and in relation to his or her goals, expectations, norms, and concerns”. More specifically, the term health-related quality of life (HRQoL) is often described as “the health-related aspects of quality of life, which are generally considered to reflect the impact of disease and treatment on disability and daily functioning” [9]. Uncontrolled asthma not only compromises the quality of life of children and their families but increases the risk of severe exacerbations, recurrent hospitalizations and even premature mortality, creating a burden on physical, emotional, social and economic levels [7,10].

Asthma treatment is not exclusively limited to the use of medication but requires a comprehensive approach that includes the control of triggering factors, education in the management of the disease—especially in the inhalation technique—and continuous follow-up, since it is a chronic condition. For the adequate control of asthma, periodic follow-up in asthma units and therapeutic education are necessary [11]. In this context, telemedicine (also known as telehealth) is presented as an innovative approach with the potential to transform healthcare. It refers to the use of technology and telecommunication systems to deliver healthcare at a distance [12,13]. The benefits are broad and transcend direct patient care. These include improved access to healthcare, early detection of asthma attacks, monitoring of adherence to treatment, reduction in hospitalizations and travel costs and better management of resources in health centers [14]. Telemedicine is easily accessible to health professionals and users and encourages participation in care through shared decision making. In general, telemedicine can be divided into three modalities: videoconferencing, remote monitoring and store and forward, which is the compilation of clinical information for subsequent transmission and analysis by a clinician. These modalities may include disease-specific symptom monitoring, treatment counseling, therapeutic education, disease self-management counseling and remote consultations with healthcare professionals. Because of the ongoing need for symptom monitoring and adequate adherence to baseline medication for disease control, asthmatic patients are ideal candidates for using these types of interventions [12,13].

Several trials have been conducted to measure the effectiveness of telemedicine throughout the world [15,16,17,18,19]. Most interventions focus on promoting self-management and ensuring adherence to treatment guidelines. Interventions include websites, mobile apps, text message reminders, store and forward, remote symptom monitoring and videoconferencing consultations. Preliminary studies suggest that these tools could improve adherence, reduce unplanned emergency department visits and empower patients and caregivers in disease self-management. Telemedicine is increasingly recognized as a valuable tool to enhance healthcare accessibility, reduce unnecessary travel and support patient follow-up across a wide range of medical conditions [17,18,19]. In pediatric asthma, telemedicine offers the possibility to reinforce self-management, monitor symptoms remotely and improve adherence to treatment, all of which are essential to achieve optimal disease control. Despite the increasing implementation of telemedicine in pediatric settings, robust and specific evidence on its clinical effectiveness in childhood asthma remains limited, as most previous reviews have focused on adult populations or included mixed-age groups without stratified analysis, making it difficult to draw firm conclusions for pediatric care. In addition, the wide variety of telemedicine modalities—ranging from synchronous video consultations to asynchronous self-management platforms—adds complexity to the assessment of their impact, which may vary depending on the context, user engagement and clinical objectives.

Although there have been meta-analyses supporting the efficacy of telemedicine in the management of asthma in adults, this evidence does not extend to the pediatric population. The lack of solid studies in this population group has motivated this review, with the aim of broadening and deepening knowledge in this field.

The objective of this review is to evaluate the clinical effectiveness of telemedicine interventions in children and adolescents with asthma. We hypothesize that telemedicine interventions are effective in improving both asthma control and quality of life in the pediatric population. For this purpose, a systematic review of the published literature will be performed by reviewing national and international studies, performed in children or adolescents, that measure the impact of telemedicine in the management of asthmatic disease, specifically in the degree of asthma control and quality of life. In addition, the different strategies used in these telehealth interventions will be described. This systematic review and meta-analysis aim to synthesize the available evidence to determine the extent of this effectiveness and to identify possible limitations and areas for future research.

## 2. Materials and Methods

The systematic review was carried out following the Preferred Reporting Items for Systematic Reviews and Meta-Analyses (PRISMA) 2020 guidelines. The protocol was previously registered in the PROSPERO (International Prospective Register of Systematic Reviews) database (ID: CRD42025251000837). Bibliographic search was performed according to the protocol published in PROSPERO. The protocol defines the search strategy, inclusion criteria, methods of information extraction and statistical analyses used in our quantitative synthesis of the literature [20]. The PRISMA guidelines were followed to present the evidence from the included studies [21]. The PRISMA checklist is shown in Supplementary Table S1.

The key questions for this study were defined using the PICO format, considering the following elements:

- Population (P): Children and adolescents (0–18 years) with a medical diagnosis of asthma, with no restrictions in terms of disease severity or gender.
- Intervention (I): Interactive telemedicine interventions, including mobile applications (apps), videoconferencing, store and forward, remote monitoring or any combination of these tools. Interventions based exclusively on telephone communication were excluded.
- Comparison (C): Usual care received by asthmatic patients according to standard clinical practice.
- Outcomes (O): Changes in quality of life and asthma control attributable to the telehealth intervention, measured at defined time points.

From these components, the following key questions were raised:

1. Do interactive telehealth interventions improve the quality of life of children and adolescents with asthma compared to usual clinical care?
2. Do these interventions contribute to better disease control in children and adolescents with asthma compared to routine clinical care?

### 2.1. Literature Search Strategy

The literature search was conducted on 11 March 2025 using Pubmed, Web of Science and Cochrane electronic databases. Specific keywords related to the topic of interest and modified to suit the characteristics of each database were included. The search strategy used is shown in Table 1. Search functions such as MeSH terms, text words, logical operators and truncated searches were used appropriately. No additional filters were added, and the time period was not limited. Subsequently, article selection was performed in two phases: reading titles and abstracts and then reading the full text. Each phase was performed independently by two reviewers, and discrepancies were resolved by consensus or with the participation of a third reviewer.

### 2.2. Literature Inclusion and Exclusion Criteria

The inclusion criteria were (1) studies aimed at pediatric and adolescent patients (0–18 years) with a diagnosis of asthma; (2) studies where patients received an interactive telemedicine intervention to help them control or live with their asthma; (3) studies reporting at least one previously established intervention outcome (degree of asthma control or quality of life); and (4) randomized clinical trials (RCTs).

The exclusion criteria were (1) studies where the intervention was exclusively via telephone; (2) studies that were not original research; (3) studies not published in English, French or Spanish; (4) studies published only in abstract form; (5) studies with inadequate control groups (without the usual care comparator or without a control group); (6) unfinished studies; and (7) meta-analyses and systematic reviews.

### 2.3. Study Selection and Data Extraction

The removal of duplicate articles was completed using the Zotero reference management software. The titles and abstracts were reviewed for eligibility criteria independently by two investigators, both pediatricians and experienced in asthmatic pathology (Moreno-Galarraga L. and Ortúzar-Menéndez M.), using the Rayyan web application. Discrepancies were resolved by consensus together with a third investigator (García-Gerriko J.) Full-text copies of the studies identified via the initial screening were obtained, and a review of the selected articles was performed to confirm alignment with the inclusion and exclusion criteria described previously. One author (García-Gerriko J.) extracted the data from the included studies, and a second author (Moreno-Galarraga L.) verified the extracted data. An ad hoc data extraction table was designed. The data extracted from the eligible articles included a description of all those items (the study method, the participants, the telemedicine intervention and the comparator), the instrument used to measure QoL, QoL outcomes, the instrument used to measure the degree of asthma control, asthma control outcomes and (if available) other outcomes of the intervention included in the study.

The risk of bias of the included randomized controlled trials was assessed using the Cochrane Risk of Bias 2.0 (RoB 2.0) tool, which evaluates five domains: (a) bias arising from the randomization process, (b) bias due to deviations from the intended interventions, (c) bias due to missing outcome data, (d) bias in the measurement of the outcome and (e) bias in the selection of the reported result [22]. A summary table detailing the risk of bias judgments for each included study, along with brief justifications, is provided as Supplementary Table S2, and as indicated, most articles were overall rated as “Low-Risk”.

### 2.4. Information Synthesis

The main objective of this review was to evaluate the effectiveness of telemedicine interventions in asthma control and quality of life. To this end, changes in the results of validated questionnaires—specific for quality of life and asthma control—before and after the telemedicine intervention were compared.

The Pediatric Asthma Quality of Life Questionnaire (PAQLQ) and Asthma Control Test/Childhood-Asthma Control Test (ACT/c-ACT) questionnaires were used to quantify quality of life and asthma control, respectively.

The PAQLQ is a self-administered questionnaire, developed specifically to measure the quality of life in asthmatic children. It contains 23 items that measure asthma-related problems that patients themselves consider affecting their daily lives and employs the same words that children use to describe their health problems. The items are distributed in three dimensions: symptoms (10 items), emotional function (8 items) and activity limitation (5 items). The response options for each PAQLQ item go from 1, indicating maximum HRQoL affection, to 7, indicating no affection [23].

ACT and c-ACT are validated questionnaires for asthma control in children. More specifically, the c-ACT is used for children aged 4–11 years, and the ACT is used for children aged 12 years and older. The c-ACT consists of seven questions that collect information from both the child and the caregiver. For the child, there are four questions with responses on a Likert scale, defined with words and pictures of a child’s face. For the caregiver, there are three questions with answers on a Likert scale, defined only with words. The c-ACT score can range from 0 (worst possible asthma control) to 27 (optimal asthma control). In both questionnaires, the child is considered to be poorly controlled if the score is below 20 [24,25].

We calculated the mean differences for each measure between the group receiving the telemedicine intervention and the group receiving usual care. We then contrasted these differences between the two groups to determine whether the observed improvement was significantly greater in the intervention group. In trials that directly reported the pre-/post-intervention mean difference, we directly extracted the value of the difference accompanied by its measure of dispersion (confidence interval). In studies that did not directly report the mean difference between groups in the pre/post change but did report the means and standard deviations before and after the intervention, this difference was calculated (pre/post mean difference of the intervention group minus the pre/post mean difference of the control group). [26]. In studies where the means of the pre- and post-intervention measurements were not provided, quantitative meta-analysis was not performed. The lack of these data made it impossible to accurately estimate the treatment effect and its errors. Therefore, we chose to include these studies only in the qualitative analysis.

A meta-analysis was performed to statistically combine the results of the selected studies, with the aim of obtaining a more accurate overall estimation of the effect of an intervention. For this purpose, comparable data (mean differences of the described questionnaires) were extracted and analyzed by means of random-effects statistical models. Analyses were performed with Stata−14.0 software, using the metan command. The result is presented graphically in a forest plot, where each study is represented by a line with its point estimate (square) and its confidence interval (horizontal line). The area of each central square is proportional to the weight of the study in the meta-analysis. Two vertical lines are presented: a continuous line corresponding to the null value (mean difference = 0) and a dashed line representing the pooled estimate (calculated mean difference) resulting from the meta-analysis. Lastly, the pooled effect (rhombus) is shown, which summarizes the results of all included studies.

Heterogeneity was calculated using the I-squared (I^2^) statistic, which quantifies the degree of heterogeneity on a single intuitive scale and is therefore comparable for any meta-analysis. It describes the percentage of the total variability between studies that is due to heterogeneity. Thus, I^2^ can be understood as a measure of the degree of heterogeneity that moves on a continuous scale ranging from 0 to 100%, where no heterogeneity is denoted by I^2^ = 0% and high heterogeneity by I^2^ > 75%. In cases of high heterogeneity, a leave-one-out sensitivity analysis was performed to assess the robustness of the meta-analysis results.

Funnel plots were also performed to detect publication bias, the main threat to the validity of a meta-analysis, since published studies may differ systematically from unpublished ones. Each point represents a study; the horizontal axis shows the estimated effect, and the vertical axis shows the precision of the study (1/SE). Ideally, the studies are symmetrically distributed in an inverted funnel shape. An asymmetry in the graph may indicate the absence of negative studies or small sample sizes, suggesting publication bias.

To ensure methodological rigor, the quality of this systematic review was independently assessed using the AMSTAR 2 (2017) (A MeaSurement Tool to Assess Systematic Reviews), a validated instrument designed to appraise the methodological quality of systematic reviews of randomized controlled trials [27]. This tool evaluates 16 key domains, including protocol registration, literature search strategy, risk of bias assessment and synthesis methods. The results of the AMSTAR 2 evaluation indicated a moderate overall level of confidence in the review’s findings. A full breakdown of the AMSTAR 2 assessment is provided as Supplementary Materials. As detected, the systematic review has more than one weakness but no critical flaws; therefore, according to AMSTAR 2 evaluation, it can provide an accurate summary of the results of the studies included (Supplementary Table S3).

## 3. Results

**Figure 1:**
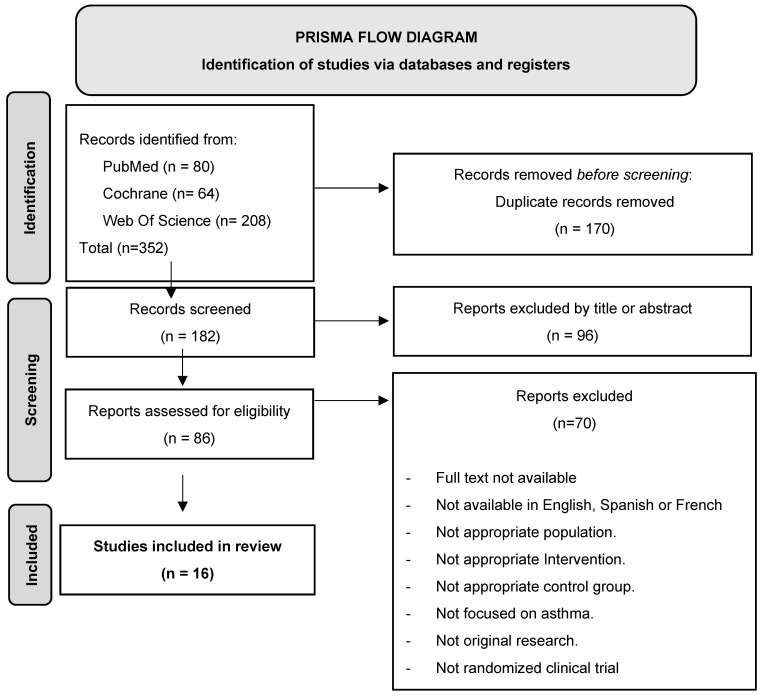
PRISMA flow diagram.

We identified 352 potentially relevant records, of which 170 were eliminated because they were duplicates. Of the remaining articles, 96 were eliminated in a first screening by title and abstract and 70 after evaluation of the full text. Thus, 16 publications met all the established criteria and were included in the analysis. Figure 1 shows the PRISMA flow diagram describing the study selection process.

### 3.1. General Characteristics of the Literature

The 16 publications, all randomized clinical trials (RCTs), included a total of 2642 participants, with sample sizes ranging from 10 to 395 patients. Of these, 1413 were in the intervention group and 1229 in the control group. Table 2 summarizes the comparative characteristics of the included RCTs. Only studies that directly reported data on mean differences were included in the meta-analysis. Although all selected studies were assessed descriptively, only those that provided specific mean difference values using the selected standardized questionnaires for asthma control or quality of life were considered for quantitative synthesis. Studies that did not report these data or used non-standardized instruments were excluded from the meta-analysis and analyzed qualitatively.

The intervention duration was 12 months in five studies [28,29,30,31,32], 6 months in five studies [33,34,35,36,37], 8 months in one study [38], 4 months in one study [39], 3 months in three studies [40,41,42] and 3 weeks in one study [43]. The age of the patients ranged from 2 to 18 years, with a mean of approximately 12 years. Only one study focused on the early childhood population (2–6 years) [31]. Only five studies reported on the severity of asthma in their subjects; of these, two included both mild and severe patients [32,42], two enrolled patients with moderate to severe asthma [29,41], and only the study by Deschildre et al. [30] included exclusively patients with severe asthma.

**Table 2:** Descriptive comparative table summarizing the general characteristics of the randomized controlled trials included in the study.

Study	Country	Number of Patients (IV/CT)	Target Population	Description from Intervention and Control	Asthma Severity	Duration
Gümüs et al. (2024) [33]	Turkey	97 (47/50)	7–17 years with a diagnosis of asthma	IV: Education through Zoom and videos on the correct use of inhalers. Daily recording of symptoms and EF values. Continuous Zoom support. CT: Usual care.	No record	6 months
Suvarna et al. (2024) [40]	India	192 (96/96)	7–17 years with a diagnosis of asthma	IV: Mobile WhatsApp from the clinic. CT: In-person visit.	No record	3 months
Shdaifat et al. (2022) [41]	Jordan	90 (45/45)	5–11 years with asthma not controlled	IV: Video call education sessions with a pharmacist. CT: Usual care.	Moderate–Severe	3 months
Fedele et al. (2021) [39]	USA	33 (17/16)	12–15 years with asthma badly controlled	IV: App designed to improve asthma management and communication with caregivers by setting goals and identifying areas for improvement. CT: Autonomous control of asthma after receiving asthma information leaflets.	No record	4 months
Kosse et al. (2019) [34]	Netherlands	234 (87/147)	12–18 years with at least two prescriptions of ICS + LABA in the last 12 months	IV: App that includes a weekly disease control test, educational videos, medication reminder and chat with the pharmacist and other patients. CT: Inhalation instructions at first dispensation.	No record	6 months
Perry et al. (2018) [35]	USA	363 (180/183)	7–14 years old with asthma	IV: Asthma telematics education for the child, caregivers and school nurse. Follow-up of symptoms and lung function performed at school. CT: Usual care.	No record	6 months
Halterman et al. (2018) [38]	USA	395 (199/199)	3–10 years with a diagnosis of asthma	IV: Mobile telemedicine unit collecting information on patients’ symptoms and physical examination. A clinician undertakes the visit from their office and conducts a video call with the caregiver. CT: Usual care.	No record	8 months
Johnson et al. (2016) [43]	USA	89 (46/43)	12–17 years with prescribed medication for asthma control	IV: App for registration and SMS medication reminders. CT: Usual care.	No record	3 weeks
Voorend-van Bergen et al. (2015) [28]	Netherlands	272 (183/89)	4–18 years with atopic asthma	IV: Monthly monitoring through a website and treatment adjustment via e-mail. CT: Usual care.	No record	12 months
Rikkers- Mutsaerts et al. (2012) [30]	Netherlands	90 (46/44)	12–18 years with a diagnosis of asthma	IV: Online self-management system. Allows weekly recording of asthma control and lung function, and patient receives instant algorithm-based feedback. CT: Usual care.	Moderate–Severe	12 months
Deschildre et al. (2012) [31]	France	50 (25/25)	6–16 years with uncontrolled allergic asthma	IV: Daily home telemonitoring of spirometry with subsequent analysis by a physician, who adjusts treatment. CT: Usual care.	Severe	12 months
Eakin et al. (2012) [30]	USA	322 (245/77)	2–6 years with a diagnosis of asthma	IV: Mobile clinic that brings asthma screening, evaluation and treatment services to schools. Information is sent to the referring physician, who reviews treatment. CT: Usual care.	No record	12 months
Xu et al. (2010) [36]	Australia	121 (80/39)	3–16 years with asthma badly controlled	IV: Interactive voice response (IVR) system. Automated telephone calls that ask questions about symptoms and medication use and provide educational messages. CT: Usual care.	No record	6 months
Chan et al. (2007) [32]	USA	120 (60/60)	6–17 years with persistent asthma	IV: Virtual visits for education. Programmed dissemination of video recordings demonstrating inhaler use and peak flow measurement. Electronic diary for recording symptoms and communicating with the physician. CT: Regular care.	Mild–Severe	12 months
Jan et al. (2007) [42]	Taiwan	164 (88/76)	6–12 years with persistent asthma	IV: An interactive online system that provides educational information, an electronic diary, an action plan, and tools for analyzing symptoms and peak flow variability CT: Verbal information and educational brochure along with a symptom log diary.	Mild–Severe	3 months
Chan et al. (2003) [37]	USA	10 (5/5)	6–17 years with persistent asthma	IV: Video review system for inhaler use and symptom control, as well as education on inhaler techniques. CT: Non-synchronous access to healthcare professionals.	No record	6 months

IV, Intervention; CT, Control; EF, Expiratory Flow; ICS, Inhaled Corticosteroid; LABA, Long-Acting Beta Agonists; FACI, Facilitated Communication Intervention in Asthma; IVR, Interactive Voice Response.

### 3.2. Characteristics of Telemedicine Interventions

The telemedicine interventions described in the sixteen studies present a wide variety of technological approaches, forms of implementation and levels of professional involvement, always adapted to the management of asthma in pediatric and adolescent populations. A considerable number of studies [32,33,34,35,37,41] employed videoconferencing, either through platforms such as Zoom or through other video call systems, to conduct disease management reviews or virtual visits and to deliver asthma education sessions; in another group, mobile applications were used that allowed symptom recording, reminders and immediate feedback through decision algorithms [34,39,43]. Other studies [29,30,32,39] implemented online monitoring and self-management systems, which facilitate periodic follow-up through the transfer of data, such as lung function, and allow treatment adjustments through asynchronous communication via e-mail or web platforms. Moreover, the study by Suvarna et al. [40] specifically evaluated the use of WhatsApp, and Xu et al. [36] used an interactive voice response (IVR) system that automates data collection and the delivery of educational messages. On the other hand, two studies [31,38] highlighted the use of mobile units in schools that combined physical data collection with real-time teleconsultations. In contrast, the control groups were predominantly limited to usual care, consisting of face-to-face visits, information leaflets, initial inhaler instructions or face-to-face access to health professionals, with no active follow-up components or remote personalization.

### 3.3. Effectiveness of Telemedicine Interventions

#### 3.3.1. PAQLQ Quality-of-Life Results

**Figure 2:**
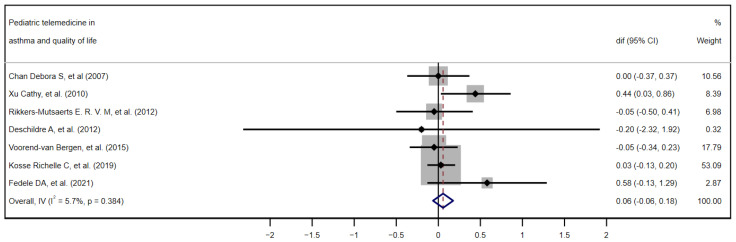
Forest plot. Effectiveness of telemedicine interventions in pediatric asthma. Quantitative meta-analysis of the seven studies included in the quality-of-life analysis in chronological order. [28,29,30,32,34,36,39] CI, confidence interval; dif, mean difference.

**Figure 3:**
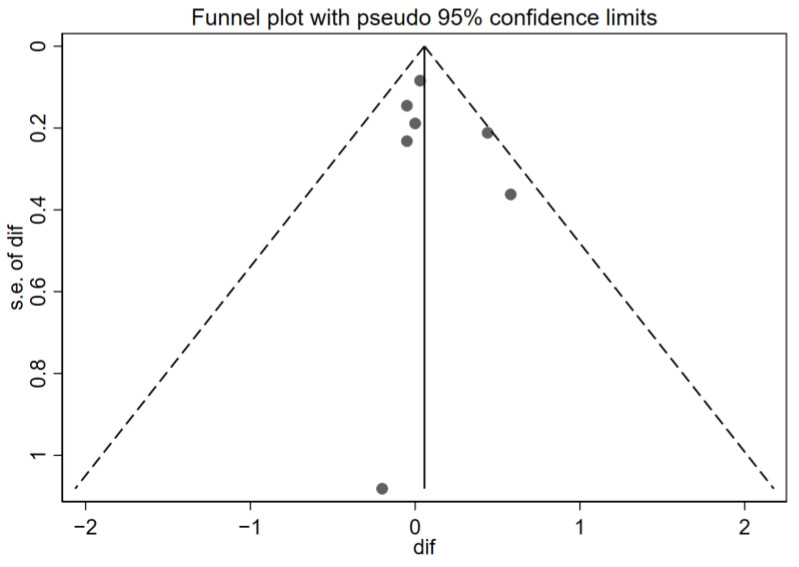
Funnel plot of the quality-of-life results. Effectiveness of telemedicine interventions in pediatric asthma.

Although all studies provided data related to the quality of life, only seven met the eligibility criteria for inclusion in the quantitative meta-analysis (the use of the Pediatric Asthma Quality of Life Questionnaire (PAQLQ) and reporting of the difference in means and standard deviations before and after the intervention). Figure 2 shows the forest plot of the quality-of-life results, ordering the seven studies chronologically. [28,29,30,32,34,36,39], and Figure 3 demonstrates the funnel plot of these same results.

Of the seven studies included, only the work of Xu et al. [36] reaches individual statistical significance (mean difference = 0.44; 95% CI 0.03 to 0.86). The study by Kosse et al. [34], with a weight of 53%, dominates the overall estimate, given its precision, followed at a distance by Voorend-van Bergen, with a weight of 17.8%. In the forest plot, the great variability in the width and position of the confidence intervals (CIs) of each study is particularly evident, reflecting differences in sample size and precision of the estimate. When applying the fixed-effects model via the inverse of variance (Overall, IV), the pooled mean difference is just 0.06 points on the PAQLQ (95% CI −0.06 to 0.18), showing a non-significant mean difference. The heterogeneity index I^2^ is very low (5.7%; *p* = 0.384), indicating that the variability between studies is minimal and that the results are essentially consistent with each other.

Additionally, inspection of the funnel plot suggests a symmetrical dispersion of the studies around the null effect line, with no clusters or gaps pointing to relevant publication bias. However, a single point well below the others stands out, corresponding to the study with the largest standard error (approx. SE = 1.0–1.2) by Deschildre et al. [30]. This difference with the rest of the points could mean that it is a potential outlier—that is, a study which, due to its results or characteristics, deviates notably from the majority of the studies included in the analysis. However, due to its extremely wide confidence interval, it has a minimal contribution to the overall result (0.32%); therefore, its presence is extremely unlikely to distort the overall conclusions.

#### 3.3.2. Other Quality-of-Life Outcomes

To complete the quality-of-life results, Table 3 presents the nine studies identified in the systematic search, which, although measuring the quality of life of the patient or caregiver, could not be included in the quantitative meta-analysis because they did not meet any of the inclusion criteria.

#### 3.3.3. ACT/c-ACT Asthma Control Outcomes

**Figure 4:**
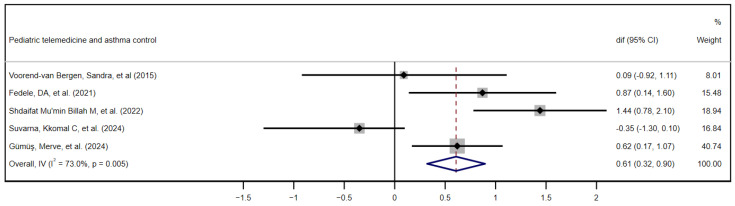
Forest plot of asthma control outcomes. Effectiveness of telemedicine interventions in pediatric asthma control. Quantitative meta-analysis of the five studies included in chronological order. [28,33,39,40,41] CI, confidence interval; dif, mean difference.

**Figure 5:**
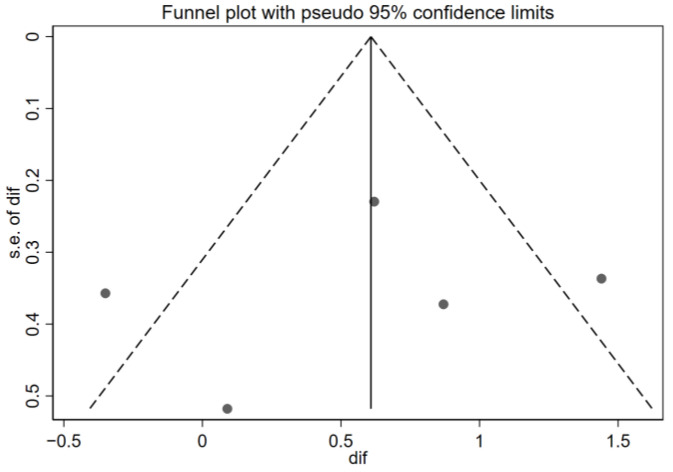
Funnel plot of the asthma control results. Effectiveness of telemedicine interventions in pediatric asthma. s.e. of dif = standard error of the mean difference.

Five studies met the eligibility criteria for inclusion in the quantitative meta-analysis. This selection was based on the application of the following requirements: first, the use of ACT or c-ACT as a standardized instrument to assess asthma control, and second, the explicit reporting of the between-group mean difference in the pre/post change or means and standard deviations before and after the intervention, as explained above. Of the five studies, two [33,41] did not directly report the difference in means between groups in the pre/post change; therefore, we calculated such a difference. Figure 4 shows the forest plot of the quality-of-life results, ordering the five studies chronologically, [28,33,39,40,41] while Figure 5 depicts the funnel plot of these same results.

The forest plot synthesizes the results of five studies evaluating the impact of telemedicine on pediatric asthma control. Among the five included investigations, three (Fedele et al. 2021: diff = 0.87, 95% CI 0.14 to 1.60; Shdaifat Mu’min Billah et al. 2022: diff = 1.44, 95% CI 0.78 to 2.10; and Gümüş et al. 2024: diff = 0.62; 95% CI 0.17 to 1.07) [33,39,41] present confidence intervals that do not cross the no-effect line, demonstrating statistically significant mean differences in favor of the telemedicine group. In contrast, Voorend-van Bergen et al. [28] and Suvarna et al. [40] show point effects very close to zero (0.09 and −0.35, respectively) with wide CIs that include the null value, evidencing the absence of statistically significant findings in those trials. The weight of Gümüş et al. (40.7%), derived from their high precision, together with moderate contributions from the studies of Shdaifat (18.9%) and Fedele (15.5%) account for a combined estimate of 0.61 (95% CI 0.32 to 0.90) under the fixed-effects model, indicating a moderate and statistically significant clinical effect of telemedicine. However, the heterogeneity index is remarkably high (I^2^ = 73.0%; *p* = 0.005), suggesting that methodological discrepancies, sample sizes and population characteristics between the studies influenced the variability in the results. Given the high heterogeneity observed in the asthma control outcome (I^2^ = 73%), a leave-one-out sensitivity analysis was conducted to evaluate the robustness of the pooled effect estimate. The results showed minimal variation when each study was excluded in turn, supporting the stability of the overall findings. The complete analysis is available in the Supplementary Materials (Supplementary Table S4).

The funnel plot shown in Figure 5 reveals a partially symmetrical distribution of studies around the overall effect line (di = 0.61), with trials on both the right side (positive effects) and left side (negative effects). The presence of dots in the lower left quadrant (small-sample negative effects) indicates that unfavorable studies were also captured, which does not support the publication bias hypothesis. However, given that there are only five studies, the visual power of the funnel plot to detect bias is limited. On the other hand, high heterogeneity (I^2^ = 73%) may distort the funnel shape, as the asymmetry could originate from both reporting biases and real variations between designs and populations.

#### 3.3.4. Other Asthma Control Outcomes

While the quantitative meta-analysis focused on the Asthma Control Test (ACT/c-ACT) scores as the primary measure of asthma control, all studies, except Johnson et al. [43], additionally provided data on objective clinical outcomes, such as inhaled and systemic corticosteroid use, number of documented exacerbations, emergency department visits and asthma-related hospitalizations. In order to provide a more complete picture of the impact of telemedicine interventions, these indicators are summarized in Table 4 below.

### 3.4. Subgroup Analysis and Sensitivity Analysis

Subgroup analysis and sensitivity analysis were not performed in this study due to the small number of studies included in each meta-analysis.

## 4. Discussion

This systematic review and meta-analysis assessed the effectiveness of telemedicine interventions in pediatric asthma. It included 16 RCTs with a total of 2642 participants. The telemedicine interventions included varied widely across studies. The meta-analysis showed significant findings on the efficacy of telemedicine in pediatric asthma control, evidenced by a significant increase in the ACT/c-ACT scores (pooled mean difference: 0.61; 95% CI: 0.32 to 0.90). However, the improvement in quality of life measured by PAQLQ was minimal and not statistically significant (mean difference = 0.06 points; 95% CI: –0.06 to 0.18).

In the analysis of the quality of life (QoL), telemedicine’s positive effects were more consistent among caregivers than among pediatric patients. A clearer trend of improvement regarding asthma control was seen in the intervention group. Recent studies like Shdaifat et al. [40] and Gümüs et al. [32] reported reductions in rescue medication use, unscheduled visits, hospitalizations and peak expiratory flow variability. Our findings align with the prior literature. A 2019 adult meta-analysis showed beneficial effects of telemedicine on asthma control (SMD = 0.78; 95% CI: 0.56–1.01) and quality of life (SMD = 0.59; 95% CI: 0.31–0.88) [43]. However, a 2021 meta-analysis with age-based sub analysis found no significant effects on quality of life in patients under 18 [17]. Similarly, a 2018 Korean pediatric meta-analysis found no significant improvement in asthma control or quality of life with telemedicine [13]. These different results between children and adults might be due to the fact that adults have greater autonomy in disease management and clearer perception of health improvements and due to the greater value adults place on telehealth’s convenience—such as avoiding travel or time off work—which is less relevant for children. These results support integrating telemedicine as a complement to in-person pediatric asthma care. Synchronous (video calls) and asynchronous (apps, websites) models can be implemented in pediatric pulmonology and primary care to monitor asthma control and adherence, especially in rural or difficult-to-reach areas [44]. Mobile clinics in schools have also proven feasible and effective in socioeconomically vulnerable settings [44]. A hybrid approach alternating virtual and in-person visits may provide optimal clinical control and patient satisfaction. Interventions should prioritize symptom monitoring, continuous education and two-way communication with healthcare providers; these three elements were present in the studies showing greater asthma control improvement [31,37,39]. Finally, the COMETA project [45] demonstrates that multidisciplinary approaches involving nursing and community pharmacies can also be effective in pediatric asthma and help optimize resources. Stratified analyses could not be performed due to the limited sample sizes and the heterogeneity of the included studies. However, after reviewing the articles individually, it appears that gender does not significantly influence the outcomes, while age may play a more relevant role, with greater impact observed in older patients. Several studies assessed quality of life both in pediatric asthma patients and their caregivers, and in many cases, improvements were more evident in caregivers than in the children themselves. Additionally, it was not possible to evaluate the effect of other variables, as many studies did not report essential clinical or sociodemographic data, such as asthma severity, type of treatment or socioeconomic background.

Among the strengths of this review are the implementation of a comprehensive systematic search that identified 352 potential records, the application of the PRISMA methodology to ensure transparency and reproducibility of the process, the prior registration of the protocol in PROSPERO, the exclusive inclusion of randomized controlled trials with broad geographic and temporal diversity, the use of RoB2 and AMSTAR 2 tools and the evaluation of two standardized outcomes (PAQLQ and ACT/c-ACT). However, several limitations must also be considered. First, the meta-analysis on asthma control showed considerable heterogeneity (I^2^ = 73%), indicating substantial variability among the included studies. Second, although 16 relevant studies were identified, not all could be included in the quantitative meta-analysis of quality of life and asthma control. Third, the meta-analysis was unable to assess the influence of potentially relevant clinical and sociodemographic variables, such as asthma severity, type of treatment or the patient’s socioeconomic background. Due to limited sample sizes and the lack of consistent reporting of these variables across studies, stratified analyses could not be performed. Future research should consider exploring these factors in greater depth, as aspects such as social vulnerability, distance to the healthcare center or the type and stage of asthma may significantly influence the effectiveness and acceptance of telemedicine interventions in pediatric populations.

Fourth, the small number of studies included in the meta-analysis prevented the performance of subgroup or sensitivity analyses. Lastly, a major limitation is the small sample sizes in many of the included studies.

Despite these limitations, the findings support the potential of telemedicine as a complementary tool in the management of pediatric asthma, particularly for follow-up, symptom monitoring and improving adherence to treatment. Considering the findings and the identified limitations, we propose several future research directions. Multicenter trials in pediatric populations are needed, with larger sample sizes, long-term follow-up and greater methodological homogeneity. Adherence to telemedicine interventions, socioeconomic factors, access to technology and distance to healthcare facilities are likely effect modifiers and should be systematically included in future studies to better assess the real-world impact of telemedicine. Also, the development of specific studies in different countries/populations would be particularly valuable to adapt these interventions to different healthcare systems and population characteristics. Likewise, it would be advisable to place greater emphasis on exploring the subjective experiences of patients, caregivers and healthcare professionals in relation to the use of telemedicine technologies. Although some studies included satisfaction surveys [39,40], these aspects are often addressed superficially and do not receive the methodological or analytical attention they deserve. Cost-effectiveness analyses represent another crucial area, as evidence suggests that telemedicine could generate significant savings by reducing hospitalizations and urgent care visits; therefore, cost-effectiveness analyses should be included in future interventions.

In conclusion, following our review, telemedicine emerges as a promising strategy for the management of asthma also in the pediatric population. Our review demonstrates that telemedicine interventions can help achieve better asthma control in pediatric patients. However, additional studies are needed to explore its long-term effectiveness in various settings. Its true potential will lie in integrating these tools in a balanced way, focused on the needs of the patient and their family and supported by solid protocols that ensure adherence, accessibility and continuity of care. Future research should prioritize the standardization of telemedicine interventions and include cost-effectiveness analyses to better guide implementation strategies. Based on current evidence, hybrid models of care—integrating both telemedicine and in-person visits—emerge as a particularly promising approach. Continued research is essential to evaluate the varied modalities of telemedicine and determine which formats offer the greatest benefit across diverse patient populations.

## Figures and Tables

**Table 1:** Search strategy based on keywords used in Pubmed, Cochrane and Web of Science.

Database	Search Terms	Articles Found
Pubmed	(“Telemedicine” [Mesh] OR “Remote Consultation” [Mesh] OR telemedicine [tiab] OR “telehealth” [tiab] OR “e-health” [tiab] OR “mobile health” [tiab] OR “mHealth” [tiab]) AND (“Pediatrics” [Mesh] OR “Child” [Mesh] OR “Infant” [Mesh] OR “Adolescent” [Mesh] OR pediatric * [tiab] OR child * [tiab] OR infant * [tiab] OR adolescent * [tiab]) AND (“Asthma” [Mesh] OR “Asthma Management” [tiab:~0] OR asthma [tiab] OR “asthma symptoms” [tiab] OR “asthma control” [tiab] OR “asthma exacerbations” [tiab])AND (“Quality of Life” [Mesh] OR “Patient Reported Outcome Measures” [Mesh] OR “Health Status” [Mesh] OR “QoL” [tiab] OR “quality of life” [tiab] OR “health-related quality of life” [tiab] OR HRQoL [tiab])	80
Cochrane	(telemedicine OR “remote consultation” OR telehealth OR “e-health” OR “mobile health” OR mHealth) AND (pediatric * OR child * OR infant * OR adolescent *) AND (asthma OR “asthma management” OR “asthma symptoms” OR “asthma control” OR “asthma exacerbations”) AND (“quality of life” OR “patient reported outcome measures” OR “health status” OR QoL OR HRQoL)	64
Web of Science	*TS = (“telemedicine” OR “remote consultation” OR “telehealth” OR “e-health” OR “mobile health” OR “mHealth”) AND* TS = (“pediatric*” OR “child*” OR “infant*” OR “adolescent*”) AND *TS = (“asthma” OR “asthma management” OR “asthma symptoms” OR “asthma control” OR “asthma exacerbations”) AND* *TS = (“quality of life” OR “patient reported outcome measures” OR “health status” OR “QoL” OR “HRQoL”)*	208

**Table 3:** Qualitative results for the quality of life. Effectiveness of telemedicine interventions in pediatric asthma.

Study	Measurement Used	Results
Chan et al. (2003) [37]	PAQLQ and PACQLQ	Patients did not perceive changes in their quality of life during the study. However, the quality-of-life survey scores increased among caregivers in the intervention group.
Jan et al. (2007) [41]	PAQLQ and PACQLQ	Both asthmatic children and their caregivers reported improvement in the quality of life after the trial, but only caregivers showed significant improvement.
Eakin et al. (2012) [31]	PACQLQ	No statistically significant improvements were identified in any group or at any evaluation time in the quality of life of the caregivers.
Johnson et al. (2016) [42]	Mini-PAQLQ	Quality of life increased from a mean of 5.7 to 6.3 in the intervention group compared to the control group (*p* = 0.037).
Perry et al. (2018) [35]	PedsQL 3.0 and Mini-PAQL	The PedsQL 3.0 scores showed a trend toward improvement in participants in the intervention group compared to baseline. However, this improvement did not reach statistical significance. There was no change from baseline in the mini-PAQLQ scores for either group.
Halterman et al. (2018) [38]	PACQLQ	Caregivers’ quality of life improved in both groups, but there were no significant differences.
Shdaifat et al. (2022) [41]	Mini-PAQLQ	Patients in the intervention group showed greater improvements in both the overall and individual domains of the Mini-PAQLQ.
Suvarna et al. (2024) [40]	Mini-PQLI (Pediatric Quality of Life Index)	There was a significant change in the mean scores for the Mini-PQLI in both the intervention and control groups. However, the mean difference in both groups was not significant.
Gümüs et al. (2024) [33]	PAQLQ	The quality-of-life scores of the intervention and control groups were similar at the beginning of the study. However, at the end of the study, quality of life was higher in the intervention group.

**Table 4:** Qualitative results for asthma control. Effectiveness of telemedicine interventions in pediatric asthma.

Study	Results
Chan et al. (2003) [37]	Patients achieved excellent control of their asthma during the study, with no emergency department visits, no hospitalizations and few unscheduled visits to the clinic for asthma. The excellent control was also reflected in the infrequent use of β-agonists.
Jan et al. (2007) [42]	Children in the intervention group had a significant decrease in night-time (*p* 0.028) and daytime (*p* 0.009) symptoms compared to children in the control group. There were no significant differences between groups in the change in morning and night-time PEF from baseline. There were no differences between groups or within groups in the ACT score at the end of the study.
Chan et al. (2007) [32]	Disease control was excellent in both groups. Emergency department visits and hospitalizations were infrequent. There were no differences in the use of rescue therapy between the groups or in forced vital capacity, forced expiratory volume in 1 s or forced expiratory flow in the mid-expiratory phase.
Xu et al. (2010) [36]	There was no statistically significant difference between the intervention groups in the need for healthcare or oral corticosteroid rescue at the end of the study, nor a clear pattern of improved outcomes in any of the three groups.
Eakin et al. (2012) [31]	An increase in symptom-free days was observed in the “Breathmobile” intervention group at 6 months compared to standard care. There was no significant reduction in hospitalizations or use of rescue medication in either group.
Deschildre et al. (2012) [30]	The risk of exacerbation was inversely related to age. There were no significant differences between the two groups in the number of days of treatment with systemic corticosteroids, nor were there significant changes between the two groups in the mean dose of inhaled corticosteroids or in lung function.
Rikkers-Mutsaerts et al. (2012) [29]	The results showed that self-management of asthma via the internet in adolescents with poorly controlled asthma led to a significant improvement in asthma control and lung function after 3 months compared to usual care. However, at 12 months, the beneficial effects were lost compared to the control group.
Voorend-van Bergen et al. (2015) [28]	The change in the number of symptom-free days did not differ significantly between groups, nor did daily symptoms, exacerbations, lung function or β-2 agonist use. A significant decrease in the dose of inhaled corticosteroids was achieved in the web group.
Halterman et al. (2018) [38]	Children in the intervention group had more symptom-free days after the intervention than children in the control group. They also had fewer daytime and night-time symptoms and fewer days with activity limitation. More children in the intervention group were prescribed preventive medication and had fewer emergency room visits than children in the control group, as well as a greater reduction in FeNO level.
Perry et al. (2018) [35]	Symptom-free days in the previous 2 weeks improved for both groups, with no statistically significant differences between them. There were no significant differences in medication prescription.
Kosse et al. (2019) [34]	The percentage of patients with adequate asthma control increased in both groups, with no significant difference between groups.
Fedele et al. (2021) [39]	FEV1 values increased in both groups but the difference between groups was not statistically significant
Shdaifat et al. (2022) [41]	All participants in the intervention group showed a significant decrease in healthcare resource utilization compared to those in the control group regarding the following parameters: number of hospitalizations, SABA refills, asthma exacerbations, SABA nebulizations/month, emergency room visits and use of corticosteroids.
Suvarna et al. (2024) [40]	There were no significant differences in the number of exacerbations.
Gümüs et al. (2024) [33]	Children in the virtual care group had fewer days with PEF levels below 80%. Children in the intervention group had lower PEF variability than children in the control group. Children in the intervention group also took less rescue medication and had more symptom-free days than children in the control group. Children in the control group had a higher number of unscheduled hospital visits.

## Data Availability

The original contributions presented in the study are included in the article/Supplementary Material, further inquiries can be directed to the corresponding author.

## Supplementary Materials

The following supporting information can be downloaded at: https://www.mdpi.com/article/10.3390/children12070849/s1.
