# Supplementary material for: Impact of Telemedicine on Asthma Control and Quality of Life in Children and Adolescents: A Systematic Review and Meta-Analysis
Source: Children (Basel). 2025 Jun 27;12(7):849. doi: 10.3390/children12070849 (PMC12293541; doi:10.3390/children12070849)
Supplement: Supplementary file 1 [file children-12-00849-s001.zip › v2_Suppl. Table S2_RoB2_Assessment.pdf]

RoB 2.0 Risk of Bias Assessment for Included RCTs

Impact of telemedicine on asthma control and quality of life in children and adolescents: a systematic review and meta-analysis

Abbreviations used in table are based on the individual domains assessed using the Cochrane Risk of Bias tool: the RoB 2 tool.

1. Randomization Process → Random.
2. Deviations from Intended Interventions → Deviations
3. Missing Outcome Data → Miss. Data
4. Measurement of the Outcome → Outcome
5. Selection of Reported Results → selection of RR
6. Overall Bias Judgment → Overall

Study (Author, Year)	Random.	Deviations	Missing Data	Outcome	Selection of RR	Overall
Chan et al., 2003 -	Low Risk	Low Risk	Low Risk	Low Risk	Low Risk	Low Risk
Chan et al., 2007 -	Low Risk	Low Risk	Low Risk	Low Risk	Low Risk	Low Risk
Deschildre et al., 2012 -	Low Risk	Low Risk	Some Concerns	Low Risk	Low Risk	Some Concerns
Eakin et al., 2012 -	Some Concerns	Some Concerns	High Risk	Low Risk	Low Risk	High Risk
Fedele et al., 2021 -	Low Risk	Low Risk	Low Risk	Low Risk	Low Risk	Low Risk
Gümüş et al., 2024 -	Low Risk	Low Risk	Low Risk	Low Risk	Low Risk	Low Risk
Halterman et al., 2018 -	Low Risk	Low Risk	Low Risk	Low Risk	Low Risk	Low Risk
Jan et al., 2007 -	Low Risk	Low Risk	Some Concerns	Low Risk	Low Risk	Some Concerns
Johnson et al., 2016 -	Low Risk	Low Risk	Low Risk	Low Risk	Low Risk	Low Risk
Kosse et al., 2019 -	Low Risk	Low Risk	Low Risk	Low Risk	Low Risk	Low Risk
Perry et al., 2018 -	Some Concerns	Low Risk	Low Risk	Low Risk	Low Risk	Some Concerns
Rikkers-Mutsaerts et al., 2012 -	Low Risk	Low Risk	Low Risk	Low Risk	Low Risk	Low Risk

Voorend-van Bergen et al., 2015 -	Low Risk	Low Risk	Low Risk	Low Risk	Low Risk	Low Risk
Xu et al., 2010 -	Low Risk	Low Risk	Low Risk	Low Risk	Low Risk	Low Risk

Table including the full title of each study, the overall RoB 2 risk rating, and a brief justification for the assessment provided.

Study (Author, Year, Title)	Overall ROB 2-Bias Judgment	Justification
Chan et al., 2003 - An Internet-based store-and-forward video home telehealth system for improving asthma outcomes in children	Low Risk	Randomization and outcome measurement well-described; low attrition and consistent reporting.
Chan et al., 2007 - Internet-based home monitoring and education of children with asthma is comparable to ideal office-based care	Low Risk	Clear methodology, robust randomization, and blinded outcome assessment.
Deschildre et al., 2012 - Home telemonitoring (FEV1) in children with severe asthma does not reduce exacerbations	Some Concerns	Good design but adherence and missing data reporting not fully detailed.
Eakin et al., 2012 - Asthma in Head Start children: Effects of the Breathmobile program and family communication on asthma outcomes	High Risk	Unclear randomization, some loss to follow-up, and risk of bias due to deviations.
Fedele et al., 2021 - Using Mobile Health to Improve Asthma Self-Management in Early Adolescence	Low Risk	Clear RCT design, low dropout, adequate reporting of adherence and follow-up.
Gümüş et al., 2024 - Virtual care for paediatric asthma: A randomized controlled trial	Low Risk	Comprehensive trial structure and transparent reporting across domains.
Halterman et al., 2018 - Effect of the SB-TEAM Program on Asthma Morbidity	Low Risk	Well-conducted school-based trial with robust randomization and follow-up.
Jan et al., 2007 - An internet-based interactive telemonitoring system for improving childhood asthma outcomes in Taiwan	Some Concerns	Design was solid, though some limitations in reporting outcome deviations.
Johnson et al., 2016 - The feasibility of text reminders to improve medication adherence in adolescents with asthma	Low Risk	Small sample but low risk due to strong design and outcome handling.
Kosse et al., 2019 - Effect of a mHealth intervention on adherence in adolescents with asthma	Low Risk	Randomized design with full adherence reporting and intention-to-treat analysis.
Perry et al., 2018 - Results of an asthma education program delivered via telemedicine in rural schools	Some Concerns	Randomization not clearly described; good reporting of outcomes and adherence.
Rikkers-Mutsaerts et al., 2012 - Internet-based self-management vs. usual care in adolescents with asthma	Low Risk	High-quality RCT with complete data reporting and valid randomization process.
Voorend-van Bergen et al., 2015 - Monitoring strategies in children with asthma: A randomized controlled trial	Low Risk	Well-executed with clearly stated randomization and outcome definitions.
Xu et al., 2010 - A randomized controlled trial of an IVR telephone system and specialist nurse support	Low Risk	Effective protocol, consistent implementation and reliable reporting.
