# Supplementary material for: Impact of Telemedicine on Asthma Control and Quality of Life in Children and Adolescents: A Systematic Review and Meta-Analysis
Source: Children (Basel). 2025 Jun 27;12(7):849. doi: 10.3390/children12070849 (PMC12293541; doi:10.3390/children12070849)
Supplement: Supplementary file 1 [file children-12-00849-s001.zip › v2_Suppl.Table S4 Sensitivity_Analysis_5_Studies.pdf]

Supplementary Table S4.

Sensitivity analysis of pooled effect size for asthma control outcome

Removed Study	Pooled Mean Difference	95% CI
**None (All included)**	**0.61**	**(0.32, 0.90)**
Voorend-van Bergen et al. (2015)	0.68	(0.31, 1.04)
Fedele et al. (2021)	0.58	(0.27, 0.89)
Shdaifat et al. (2022)	0.39	(0.06, 0.72)
Suvarna et al. (2024)	0.75	(0.39, 1.11)
Gümüş et al. (2024)	0.45	(0.19, 0.71)

Table footnote:

This analysis was conducted using a leave-one-out method to assess the robustness of the pooled mean difference in asthma control. The effect estimates (Mean difference and 95% CI) are presented with and without each individual study. No single study substantially altered the overall effect size.
