# Supplementary material for: Impact of Telemedicine on Asthma Control and Quality of Life in Children and Adolescents: A Systematic Review and Meta-Analysis
Source: Children (Basel). 2025 Jun 27;12(7):849. doi: 10.3390/children12070849 (PMC12293541; doi:10.3390/children12070849)
Supplement: Supplementary file 1 [file children-12-00849-s001.zip › v2_Suppl. Table S3_AMSTAR2.pdf]

AMSTAR 2 Evaluation:

Impact of telemedicine on asthma control and quality of life in children and adolescents: a systematic review and meta-analysis

**Rating overall confidence in the results of the review: MODERATE**
More than one non-critical weakness: the systematic review has more than one weakness but no critical flaws.
It may provide an accurate summary of the results of the available studies that were included in the review

1. Did the research questions and inclusion criteria for the review include the components of PICO?

Answer: Yes

Justification: The review clearly defines the Population (children with asthma), the Intervention (telemedicine), Comparator (standard care), and Outcomes (asthma control and quality of life). This aligns with PICO criteria.

---

2. Did the report of the review contain an explicit statement that the review methods were established prior to the conduct of the review and did the report justify any significant deviations from the protocol?

Answer: Yes

Justification: The review states that the protocol was registered in PROSPERO (ID: CRD42023453265). No protocol deviations are reported, which is acceptable if none occurred.

---

3. Did the review authors explain their selection of the study designs for inclusion in the review?

Answer: Yes

Justification: The authors justify including only randomized controlled trials (RCTs), consistent with the aim of evaluating intervention effectiveness.

---

4. Did the review authors use a comprehensive literature search strategy?

Answer: Yes

Justification: The search strategy includes multiple databases (PubMed, Scopus, Embase, Web of Science, Cochrane CENTRAL) and uses MeSH terms and Boolean operators. Search limits and filters are explained.

---

5. Did the review authors perform study selection in duplicate?

Answer: Partial Yes

Justification: The review mentions that two authors were involved in screening, but it is unclear whether this was done independently and in duplicate at all stages.

---

6. Did the review authors perform data extraction in duplicate?

Answer: Partial Yes

Justification: Data extraction was performed by more than one author, but it is not explicitly stated that this was done independently and in duplicate.

---

7. Did the review authors provide a list of excluded studies and justify the exclusions?

Answer: No

Justification: There is no appendix or section listing excluded studies with reasons for exclusion.

---

8. Did the review authors describe the included studies in adequate detail?

Answer: Yes

Justification: The included studies are thoroughly described in a detailed table, including study design, population, intervention, outcomes, and follow-up period.

---

9. Did the review authors use a satisfactory technique for assessing the risk of bias (RoB) in individual studies?

Answer: Yes

Justification: The review uses the Cochrane RoB 2.0 tool, applied and reported consistently for all included studies.

---

10. Did the review authors report on the sources of funding for the studies included in the review?

Answer: No

Justification: There is no indication that the authors assessed or reported the funding sources of the primary studies. (but authors did provide a report on the sources funding their study)

---

11. If meta-analysis was performed, did the review authors use appropriate methods for statistical combination of results?

Answer: Yes

Justification: A random-effects model is used appropriately. Statistical heterogeneity (I^2), Funnel plots and sensitivity analyses are presented.

---

12. If meta-analysis was performed, did the review authors assess the potential impact of RoB in individual studies on the results of the meta-analysis or other evidence synthesis?

Answer: Partial Yes

Justification: The discussion mentions limitations in study quality, but no stratified analyses were performed based on RoB levels.

13. Did the review authors account for RoB in individual studies when interpreting/discussing the results of the review?

Answer: Yes

Justification: The authors explicitly discuss how limitations in individual studies might affect the validity of the results.

---

14. Did the review authors provide a satisfactory explanation for, and discussion of, any heterogeneity observed in the results of the review?

Answer: Yes

Justification: Heterogeneity is evaluated and potential sources (e.g., differences in age groups and intervention types) are considered in the discussion.

---

15. If quantitative synthesis was performed, did the review authors carry out an adequate investigation of publication bias and discuss its likely impact on the results of the review?

Answer: Partial Yes

Justification: Publication bias is briefly acknowledged, and also funnel plot are reported.

---

16. Did the review authors report any potential sources of conflict of interest, including any funding they received for conducting the review?

Answer: Yes

Justification: The authors clearly state that there were no conflicts of interest and no funding received for this work.
